# Association between social support and ambulance use among older people in Japan: an empirical cross-sectional study

**DOI:** 10.1186/s12873-024-00953-8

**Published:** 2024-03-05

**Authors:** Yotaro Asano, Tomo Takasugi, Keiko Ueno, Naoki Kondo, Atsuto Yoshino, Toshiyuki Ojima

**Affiliations:** 1 Department of Community Health and Preventive Medicine, Hamamatsu University School of Medicine, Shizuoka, Japan; 2 Center for Preventive Medical Sciences, Chiba University, Chiba, Japan; 3 Department of Social Epidemiology, Graduate School of Medicine and School of Public Health, Kyoto University, Kyoto, Japan; 4 Department of Medicine Emergency & Disaster Medicine, Hamamatsu University School of Medicine, Shizuoka, Japan

**Keywords:** Social support, Ambulance, Emergency department, Japan Gerontological Evaluation Study, Family support, Social prescribing

## Abstract

**Background:**

Ambulance service demand and utilization are increasing worldwide. To address this issue, the factors that affect ambulance use must be identified. Few studies have examined factors that can intervene and thus reduce the frequency of ambulance use. This study aimed to examine the association between social support and ambulance use among older adults in Japan. We hypothesize that social support is associated with reduced ambulance use.

**Methods:**

This cross-sectional study was conducted as part of the Japan Gerontological Evaluation Study. In December 2019 and January 2020, we collaborated with individuals aged 65 years or above with no long-term care needs. A total of 24,581 participants were included in the analysis. The objective and explanatory variables were ambulance use and social support, respectively. Binomial regression analysis was used to calculate the odds ratios (ORs) and 95% confidence intervals (CIs).

**Results:**

Social support was associated with ambulance use. People who had no one to listen to their complaints or worries were significantly more likely to use ambulance services than those who did (OR [95% CI] = 1.26 [1.03–1.53]). People with no one to take care of them when they were ill were also significantly more likely to use ambulance services than those who had someone to provide care (1.15 [1.01–1.31]). Moreover, the results of binomial logistic regression analysis indicated that individuals who called an ambulance but were not hospitalized had significantly lower social support compared to those who did not call an ambulance.

**Conclusions:**

The results suggest that the presence and quality of social support play a significant role in ambulance use among older adults in Japan. Our findings can help policymakers to plan and implement strategies for reducing the burden on emergency medical care.

**Supplementary Information:**

The online version contains supplementary material available at 10.1186/s12873-024-00953-8.

## Background

In emergency medicine, a worldwide increase in the demand for ambulances and ambulance utilization has become a critical issue [1–7]. For example, the demand for ambulances in the United Kingdom increased by approximately 4% per year for almost a decade from 2010 [1]. Ambulance utilization is particularly high among older adults who use the service for relatively non-urgent problems because they have multiple health problems [8]. Currently, there is no effective solution for this issue [3], which carries individual and societal costs. For example, in the United States, the costs of ambulance services are charged to the patients or insurance companies; while in Japan, anyone can use ambulance services at no financial charge by dialing 119. In other words, local governments defray the full operational costs in Japan and provide ambulance use as a public service. Therefore, as the demand for ambulance services increases, the cost to local governments also increases [9]. Therefore, it is important to identify the factors related to ambulance utilization among older adults.

Situations in which patients use the ambulance but are not admitted to the hospital include mild medical conditions in which the patients think an ambulance is needed, but medical staff consider the call for an ambulance inappropriate; these situations indicate that patients often have difficulty determining what circumstances require calling an ambulance. For example, some studies have reported that non-urgent medical visits may cause crowding in emergency departments [10]. Therefore, clarifying the factors of ambulance use that do not require medical intervention may help reduce the overall demand and, thereby, reduce congestion at emergency departments.

Older adults, males, and people who have a lower annual income have been associated with a high frequency of ambulance use [2, 5, 11–15]. For example, older adults are associated with frequent ambulance use because they are more likely to have serious diseases, such as cerebral or cardiovascular diseases [13]. However, we think that designing interventions to address factors other than annual income is challenging and the welfare system may impact income. Further, the impact of welfare system takes time and is limited.

To the best of our knowledge, few studies have examined factors that can intervene and thus reduce the frequency of ambulance use among older adults. In this context, the relationship between social support and ambulance use is important. In one study, people who arrived at the hospital by ambulance had significantly lower social support than those who arrived by their own means of transport [16]; however, as that study was conducted at a single institution, the sample size was limited [16].

Therefore, this study aimed to examine the association between social support and ambulance use among older adults in Japan. We hypothesize that social support is associated with reduced ambulance use among older people in Japan.

## Methods

### Study setting

This study was designed as a cross-sectional study. This research was conducted as a part of the Japan Gerontological Evaluation Study (JAGES). The main objectives of JAGES are to clarify health disparities, directions for care prevention strategies and the social determinants of health among people over 65 years old. The survey was conducted in cooperation with local governments that identified with the JAGES objectives and offered to cooperate. The JAGES questionnaire is based on the national daily living area needs assessment data. While using the data, experts in each field consulted with each other. They added scales whose reliability and validity were confirmed or added items if they were yet to be developed. The survey items were set from multiple perspectives, including physical, psychological, and social items. The JAGES questionnaire is continuously being revised based on the JAGES’s knowledge from the previous studies [17, 18].

The JAGES collaborated with municipal governments and mailed questionnaires to 345,356 community-dwelling people aged 65 years or older without long-term care needs. The selection of the respondents was randomized. The participants were selected from 64 municipalities, including metropolitan, urban, semi-urban, and rural areas in 24 prefectures in Japan, from Hokkaido (the northernmost prefecture in Japan) to Kyushu (the southernmost region in Japan) (Supplemental Fig. 1). The sampling of participants for the JAGES survey was done at the municipal level and was randomized. The sampling frame was based on a list of older people (65 years or older), obtained from either long-term care insurance or the basic resident register, whichever was easier for the municipality to use. The JAGES questionnaire was developed from June 2018 to October 2019. Questionnaire distribution, follow-up, and data collection were conducted from November 2019 to January 2020.

To increase the response rate, JAGES used techniques such as call center placement and distribution of thank you reminder letters. As an incentive, the researchers shared the results of data analysis with the municipality and residents.

### Participants

**Fig. 1:**
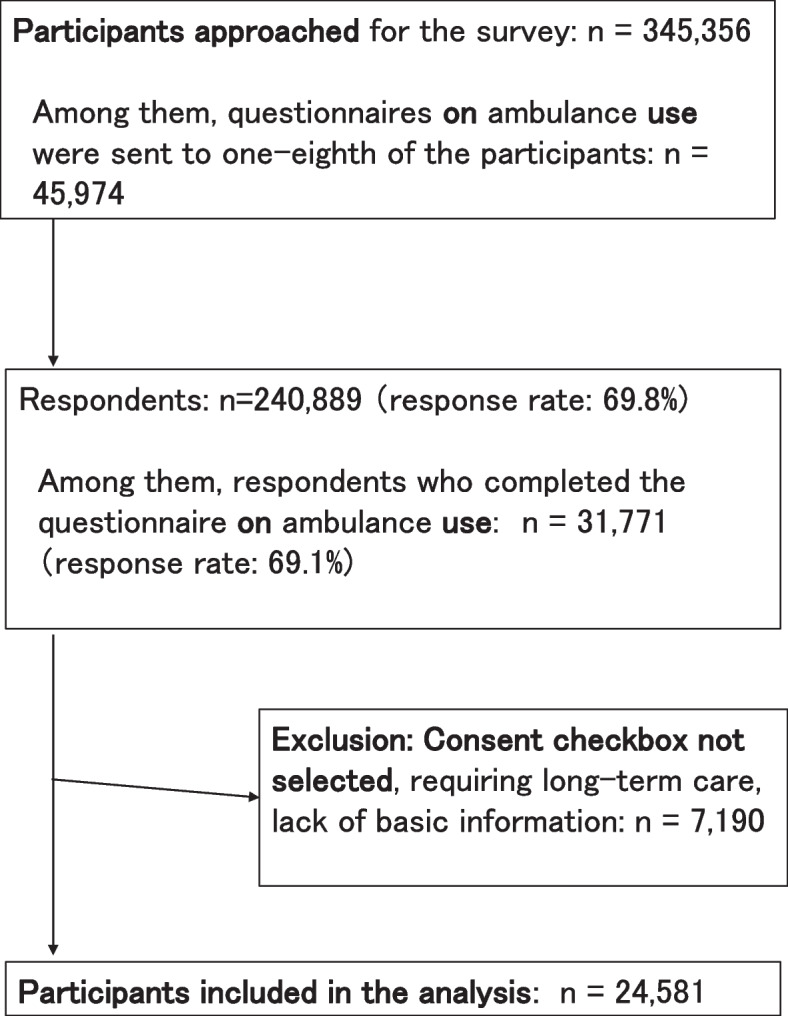
Flow of participants during the study

Figure 1 shows the process of participant selection in this study. A total of 240,889 individuals from 64 municipalities responded to the questionnaire (response rate: 69.8%, range: 54.4–89.8%). One-eighth of the participants (*n* = 45,974) were randomly selected, and a questionnaire containing items about the frequency of ambulance use was administered. A total of 31,771 people subsequently responded, and 24,581 were included in the analysis; 7190 were excluded due to failing to provide informed consent, requiring long-term care for daily living, and omitting basic information such as sex and age. This study was approved by the Ethics Committee at the National Center for Geriatrics and Gerontology in Japan (approval number: 1274–2; date: December 18, 2020), at Chiba University (approval number: 3442; date: December 11, 2019), and at Japan Gerontological Evaluation and Research Institute (approval number: 2019–01; date: October 10, 2020) and was performed in accordance with the Declaration of Helsinki.

All participants were informed that participation in this study was voluntary and that completing the questionnaire, selecting the checkbox for approval, and returning it by mail would indicate their consent to participate.

### Measures

#### Objective variable

Two outcomes were used in the binomial logistic regression analyses. The first outcome was “whether or not the participant used an ambulance “ [19]. The second outcome was “whether or not” the participant was hospitalized after using an ambulance. “ As noted in the introduction to this study, we developed the outcome that participants who used ambulances but were not hospitalized are those considered “unnecessary use“ from view of medical staff. The following questions were used to determine participants’ ambulance use and the number of hospitalizations: “Have you called an ambulance for yourself or had someone call one for you in the past year? “and “How many times have you been hospitalized after visiting a medical facility by ambulance? ““Participants answered each of these questions by selecting one of the five categories that apply to them (1–3 times, 4–6 times, 7–9 times, ≥ 10 times, or never). We dichotomized their response of ambulance call and hospitalization after ambulance transport into binary variables that exhibited never (zero times), or one or more times.

#### Explanatory variable

Participants ‘social support was examined using the following four questions: “Is there someone who listens to your worries and complaints? ““Do you have someone whose worries and complaints you listen to? ““Is there someone who takes care of you when you fall ill for a few days? “and “Do you have someone who you take care of when they fall ill for a few days? “ [20].

Participants responded to these questions with multiple answers: spouse, children living together, children living separately, siblings/relatives/parents/grandchildren, neighbors, friends, and none. For the data analysis, we categorized the responses into three categories: “family” (spouse, children living together, children living separately, and siblings /relatives/parents/grandchildren); “neighbors/friends” (neighborhood and friends); “none”.

#### Covariates

Participants were categorized by age (65–69, 70–74, 75–79, 80–84, ≥ 85 years), sex (men, women), years of education (< 6, 6–9, 10–12, ≥ 13 years), marital status (yes: currently married, no: not currently married) and self-rated health status (good, bad). Annual equivalent income was calculated by dividing household income by the square root of the number of household members and was divided into three groups (≥ 4 million yen, 2–4 million yen, or < 2 million yen per year; 1 dollar = 110 yen in 2019) [21].

#### Data analysis

We described the characteristics of all the study participants for three groups. Group 1: people who never called an ambulance. Group 2: people who called an ambulance at least once but were not hospitalized after ambulance transport. Group 3: people who called an ambulance at least once and were hospitalized at least once after ambulance transport [19]. We described the characteristics of frequent ambulance users (e.g. ≥ 10 times, 4–6 times, 7–9 times) of this study.

We conducted a binomial logistic regression analysis to examine the relationship between social support and ambulance use. First, odds ratios (ORs) and 95% confidence intervals (CIs) were calculated for people who had never called an ambulance (Group 1), as opposed to those who had called an ambulance at least once (Groups 2 and 3), to determine the characteristics of people using ambulances. Second, ORs and 95% CIs were also calculated for people who had never called an ambulance (Group 1), as opposed to people who had called an ambulance but were not hospitalized after ambulance transport (Group 2). The aim of this analysis was to clarify the use of ambulances for mild conditions or cases in which patients thought ambulance service was necessary but seemed unnecessary from the medical personnels’ perspective.

For both analyses, the following three models were applied: Model 1 included social support as a covariate; Model 2 included Model 1 and sex and age; and Model 3 included Model 2 plus health status, marital status, equivalent annual income, and years of education. In the multivariate analyses reported in Tables 2 and 3, multicollinearity was checked using variance inflation factors. Similarly, the model goodness of fit and discriminant ability was checked with the Hosmer-Lemeshow test and c-statistic, respectively.

For annual income, a missing-value category was created. For all the other variables, data with missing values were excluded from the analysis. *p*-value < 0.05 was interpreted as statistically significant for all analyses. The analyses were conducted using IBM SPSS Statistics for Windows, version 26.0 (IBM Corp., Tokyo, Japan).

## Results

Table 1 summarizes the descriptive data on ambulance use. Being male, being older, having poorer self-rated health, and having lower income were associated with hospitalization after ambulance use. Having fewer years of education and having no spouse were also associated with frequent ambulance use. Being older, having a lower income, and lacking social support were associated with hospitalization after ambulance use (Supplemental Table 1).

**Table 1:** Characteristics of study participants

	Total		People who did not call an ambulance		People who called ambulance, but were not hospitalized		People who called an ambulance and were hospitalized	
	n	%	n	%	n	%	n	%
Gender	21,471							
Female	11,191	52.1	10,499	52.6	341	50.0	350	41.5
Male	10,280	47.9	9446	47.4	342	50.0	493	58.5
Age (years)	21,471							
65 ~ 69	5155	24.0	4922	24.7	117	17.1	116	13.8
70 ~ 74	6383	29.7	6016	30.1	164	24.0	203	24.1
75 ~ 79	5162	24.0	4768	23.9	175	25.6	219	26.0
80 ~ 84	3135	14.6	2820	14.1	131	19.1	184	21.8
≧85	1636	7.6	1419	7.1	96	14.1	121	14.4
Years of education	21,068							
≧13	6486	30.8	6113	31.2	176	26.1	197	24.3
10 ~ 12	9035	42.9	8423	43.0	271	40.2	341	42.1
6 ~ 9	5233	24.8	4765	24.3	214	31.8	254	31.4
< 6	143	0.7	126	0.6	7	1.0	10	1.2
Others	171	0.8	157	0.8	6	0.9	8	1.0
Self-rated health status	21,149							
Good	18,268	86.4	172,233	98.6	499	74.3	546	66.7
Poor	2881	13.6	2435	1.4	173	25.7	273	33.4
Marital status (currently married)	21,150							
Yes	15,502	73.3	14,441	73.5	463	68.7	598	73.3
No	5483	26.0	5073	25.8	201	29.8	209	25.6
Others	165	0.8	146	0.7	10	1.5	9	1.1
Annual equivalent income	21,471							
≧4 million	2200	10.2	2111	10.6	52	76.1	57	6.8
2 million~ < 4 million	7464	34.8	7006	35.1	228	33.4	230	27.3
< 2 million	9158	42.7	8416	42.2	314	46.0	428	50.8
non-responsive	2629	12.2	2412	12.1	89	13.0	128	15.2
**About people around you who listen to your complaints**								
Family members listen to your complaints	21,122							
Yes	18,026	85.3	16,812	85.6	539	81.4	675	82.4
No	3096	14.7	2829	14.4	123	18.6	144	17.6
Neighbors or friends listen to your complaints	21,122							
Yes	10,670	50.5	10,007	51.0	319	48.2	344	42.0
No	10,452	49.5	9634	49.0	343	51.8	475	58.0
There is no one to listen to your complaints	21,122							
Yes	1028	4.9	931	4.7	54	8.2	43	5.3
No	20,094	95.1	18,710	95.3	608	91.8	776	94.7
**About people around you whose complaints you listen to**								
You listen to your family’s complaints	20,987							
Yes	16,991	81.0	15,874	81.2	500	75.6	617	77.2
No	3996	19.0	3653	18.8	161	24.4	182	22.8
You listen to neighboors’ or friends’ complaints	20,987							
Yes	10,825	51.6	10,180	52.1	316	47.8	329	41.1
No	10,162	48.4	9347	47.9	345	52.2	470	58.9
You have no one to listen to his or her complaints	20,987							
Yes	1326	6.3	1183	6.1	71	10.7	72	9.0
No	19,661	93.7	18,334	93.9	590	89.3	727	91.0
**About pople who take care of you when you fall ill**								
Family members take care of you	21,141							
Yes	19,693	93.1	18,350	93.4	601	90.0	742	90.1
No	1448	6.9	1303	6.6	68	10.0	77	9.9
Neighbors or friends take care of you	21,141							
Yes	1594	7.5	1461	7.4	54	8.1	79	9.6
No	19,547	92.5	18,192	92.6	615	91.9	740	90.4
There is no one who takes care of you	21,141							
Yes	1078	5.1	977	5.0	50	7.5	51	6.2
No	20,063	94.9	18,676	95.0	619	91.5	368	93.8
**About people who you take care of when they fall ill**								
You take care of family	21,141							
Yes	15,687	74.2	14,675	74.7	459	68.6	553	65.1
No	5080	25.8	4656	25.3	187	31.4	237	34.9
You take care of neighbors or friends	21,141							
Yes	1773	8.4	1648	8.4	51	76.2	74	9.0
No	18,994	91.6	17,683	91.6	595	23.8	716	91.0
There is no one who you take care of	21,141							
Yes	4600	21.8	4216	21.5	169	25.3	215	26.3
No	16,167	88.2	15,115	78.5	477	74.7	575	73.7

In the analysis comparing Group 1 with Groups 2 and 3, people who had no one who could attend to them for complaints or worries were more likely to make more ambulance calls (in Model 3: OR [95% CI]: 1.26 [1.03–1.53]) (Table 2). People who had no one who could take care of them during an illness were significantly associated with more ambulance use than those who had a person who could take care of them (1.15 [1.01–1.31]). Overall, people whose family members listened to their complaints were less likely to call an ambulance than those whose family members did not (0.83 [0.71–0.96]). Additionally, individuals who listened to their family members’ complaints or worries also tended to be less likely to call an ambulance than those who did not (0.85 [0.75–0.93]). Moreover, people who have family members to care of them when they are ill called ambulances less frequently compared to those who did not (0.73 [0.60–0.90]). However, people who were cared for by neighbors/friends were considerably more likely to avail themselves of ambulance services than those who were not (1.34 [1.10–1.63]). People who took care of their ill family members were significantly less likely to call ambulances than those who did not (0.81 [0.72–0.91]).

**Table 2:** Binomial logistic regression analysis of the relationship between social support and ambulance call

Variables		Categories	Model 1			Model 2			Model 3		
			OR	(95%CI)	P	OR	(95%CI)	P	OR	(95%CI)	P
**About people around you who to listen to your complaints**											
	**1**	Family members listen to your complaints	0.77	0.67 to 0.88	**0.00**	0.77	0.67 to 0.88	**0.00**	0.83	0.71 to 0.96	**0.01**
	**2**	Neigbors or friends listen to your complaints	0.78	0.70 to 0.86	**0.00**	0.89	0.80 to 0.99	**0.04**	0.97	0.86 to 1.08	0.54
	**3**	There is no one to listen to your complaints	1.40	1.13 to 1.73	**0.00**	1.27	1.02 to 1.58	**0.03**	1.11	0.88 to 1.39	0.39
**About people around you whose complaints you listen to**											
	**1**	You listen to your family’s complaints	0.76	0.70 to 0.86	**0.00**	0.78	0.69 to 0.88	**0.00**	0.85	0.75 to 0.98	**0.02**
	**2**	You listen to neighbors’ or friends’ complaints	0.72	0.64 to 0.80	**0.00**	0.82	0.73 to 0.91	**0.00**	0.90	0.80 to 1.01	0.07
	**3**	You have no one whose complaints you listen to	1.69	1.41 to 2.02	**0.00**	1.48	1.30 to 1.77	**0.00**	1.26	1.03 to 1.53	**0.02**
**About pople who take care of you when you fall ill**											
	**1**	Family members take care of you	0.64	0.54 to 0.77	**0.00**	0.63	0.53 to 0.75	**0.00**	0.73	0.60 to 0.90	**0.00**
	**2**	Neighbors or friends take care of you	1.24	1.04 to 1.49	**0.02**	1.34	1.12 to 1.62	**0.00**	1.34	1.10 to 1.63	**0.00**
	**3**	There is no one who takes care of you	1.39	1.13 to 1.72	**0.00**	1.44	1.17 to 1.77	**0.00**	1.22	0.97 to 1.54	0.09
**About people who you take care of when they fall ill**											
	**1**	You take care of your family	0.76	0.68 to 0.86	**0.00**	0.81	0.72 to 0.91	**0.00**	0.81	0.72 to 0.91	**0.00**
	**2**	You take care of neighbors or friends	1.01	0.84 to 1.22	0.92	1.10	0.91 to 1.33	0.33	1.13	0.93 to 1.38	0.22
	**3**	There is no one who you take care of	1.30	1.16 to 1.47	**0.00**	1.22	1.08 to 1.38	**0.00**	1.15	1.01 to 1.31	**0.04**

*OR* odds ratio: *CI* confidence interval

*Model 1: adjusted for social support

*Model 2: Model 1 + adjusted for age and sex

*Model 3: Model 2+ adjusted for health status, marital status, annual equivalent income and years of education

Bold P figure: *p* < 0.05

In the analysis comparing Group 1 with Group 2, social support was significantly lower among those who called an ambulance but who were not hospitalized after ambulance transport than those who did not call an ambulance (Table 3). In Model 3, the most frequent ambulance use was observed among individuals who did not attend to anyone’s’ complaints (OR [95% CI]: 1.46 [1.11–1.91]), those who had no one who listened to their complaints (1.58 [1.16–2.14], and those who had no one to take care of them when ill (1.39 [1.01–1.92]). These results were confirmed after adjusting for sex, age, health status, years of education, marital status, and income level.

**Table 3:** People who did not call an ambulance versus people who did but were not hospitalized

Variables		Categories	Model 1			Model 2			Model 3		
			OR	(95%CI)	P	OR	(95%CI)	P	OR	(95%CI)	P
**About people around you who to listen to your complaints**											
	**1**	Family members listen to your complaints	0.74	0.60 to 0.90	**0.00**	0.74	0.61 to 0.91	**0.00**	0.76	0.62 to 0.94	**0.01**
	**2**	Neigbors or friends listen to your complaints	0.90	0.77 to 1.05	0.16	0.98	0.83 to 1.15	0.78	1.05	0.90 to 1.24	0.57
	**3**	There is no one to listen to your complaints	1.79	1.34 to 2.38	**0.00**	1.74	1.30 to 2.33	**0.00**	1.58	1.16 to 2.14	**0.00**
**About people around you whose complaints you listen to**											
	**1**	You listen to your family’s complaints	0.72	0.60 to 0.86	**0.00**	0.75	0.62 to 0.90	**0.00**	0.79	0.65 to 0.96	**0.02**
	**2**	You listen to neighbors’ or friends’ complaints	0.84	0.72 to 0.98	**0.03**	0.92	0.78 to 1.08	0.28	0.99	0.84 to 1.18	0.94
	**3**	You have no one whose complaints you listen to	1.54	1.15 to 2.07	**0.00**	1.69	1.31 to 2.19	**0.00**	1.46	1.11 to 1.91	**0.01**
**About pople who take care of you when you fall ill**											
	**1**	Family members take care of you	0.63	0.49 to 0.81	**0.00**	0.62	0.48 to 0.80	**0.00**	0.70	0.52 to 0.92	**0.01**
	**2**	Neighbors or friends take care of you	1.09	0.82 to 1.45	0.54	1.11	0.84 to 1.48	0.47	1.11	0.82 to 1.50	0.49
	**3**	There is no one who takes care of you	1.54	1.15 to 2.07	**0.00**	1.60	1.19 to 2.15	**0.00**	1.39	1.01 to 1.92	**0.04**
**About people who you take care of when they fall ill**											
	**1**	You take care of your family	0.78	0.66 to 0.92	**0.01**	0.82	0.69 to 0.98	**0.03**	0.91	0.75 to 1.10	0.31
	**2**	You take care of neighbors or friends	0.92	0.69 to 1.23	0.57	0.96	0.71 to 1.28	0.77	0.96	0.71 to 1.31	0.81
	**3**	There is no one who you take care of	1.27	1.06 to 1.52	**0.01**	1.20	1.00 to 1.43	**0.05**	1.10	0.91 to 1.34	0.33

*OR* odds ratio: *CI* confidence interval

*Model 1: adjusted for social support

*Model 2: Model 1 + adjusted for age and sex

*Model 3: Model 2+ adjusted for health status, marital status, annual equivalent income and years of education

Bold P figure: *p* < 0.05

Binomial logistic regression analysis for people who did not call an ambulance versus people who did but were not hospitalized after ambulance transport

In the multivariate analyses reported in Tables 2 and 3, the results of the multicollinearity check using the variance inflation factor show that no multicollinearity was observed because all variables in models 2 and 3 had a VIF less than 10 (Supplemental Table 2). The results of the Hosmer-Lemeshow test show that most of the variables in Models 2 and 3 had *p*-values greater than 0.05, and in addition, the model fit was good, considering the positive discrimination rate. The c-statistic shows that fit rates of most of the models were poor (Supplemental Tables 3 and 4).

## Discussion

Older people who had never called an ambulance were more likely to receive family and social support, such as listening to someone’s’ complaints or taking care of someone when they get ill, than those who had called an ambulance at least once. Older people who had never called an ambulance were more likely to receive family support (except for taking care of family) and social support, having their complaints listened to, listening to someone’s’ complaints, or being cared for when ill, than those who had called an ambulance but were not hospitalized after ambulance transport.

The lack of social support was associated with a tendency to call an ambulance. Moreover, we found that social support was significantly lower among those not hospitalized after calling an ambulance. This result is consistent with that of a previous study [16]. In a study that interviewed older patients who visited the emergency department with lower clinical urgency, 66% of them reported that they were dissatisfied with their level of social interaction with others [22]. Therefore, based on the results of this study, policies to reduce unnecessary ambulance use, wherein physicians prescribe a greater provision of social support for older people who use ambulances too frequently, should be implemented to reduce the burden on emergency departments. Social prescribing is also known as “community referral;” it provides a way of linking patients in primary care to their nonmedical sources of support within the community [23]. As a practical example, we think that social prescriptions such as salons for older people may reduce ambulance use among older people.

In this study, having family support was associated with ambulance use. A lack of family support has been reported to be associated with emergency department admissions [24]. We believe that the presence or absence of family is a pertinent factor that determines ambulance use. For example, providing support for family members, approaching an agency or organization that provides support for family members, and family-like counseling for people who live alone or are estranged from their families may reduce ambulance use, and thereby the burden on emergency medicine departments.

A notable finding of this study is that older people are less likely to call an ambulance if they are taken care of by family members when they become ill; however, they are more likely to call an ambulance if neighbors or friends take care of them in the same situation. Neighbors and friends are often less familiar with their illness and symptoms than family members. This tendency can, therefore, also be expected in cases where an ambulance is requested for non-emergency health conditions. At the same time, older people who receive care from family members may go to a medical institution by themselves. However, if older people are taken care of by neighbors or friends, they may call for an ambulance. Older people whose neighbors and friends call an ambulance may benefit from medical services at home (e.g., home nursing, visiting physicians) and legal systems consulting about sudden illnesses as the number of older people living alone is increasing.

### Limitations

This study had several limitations. First, due to the cross-sectional design of the study, reverse causality exists. Therefore, future studies should expand on this study by analyzing data using a longitudinal design. Second, neither data about the diseases with which the older people were diagnosed after ambulance use nor their medication history could be collected in this study. Additionally, although “admission” vs “no admission” is a reasonable category to separate mild from severe medical conditions, some major medical conditions can result in being discharged from the emergency department, such as fractures of the extremities, head injuries, lacerations, etc. It cannot be ruled out, therefore, that this clinical information may be confounding. Hence, future studies should determine the relationship between ambulance use and social support by adjusting for disease severity, diagnosis, and medication. Third, the findings in this study may not be generalizable to all older people because of sampling bias resulting from only using older people who did not need long-term care. However, the effect of sampling bias on the result was minimal because the participants were selected randomly. Finally, external validity may be low when this study is implemented in contexts such as underdeveloped social environments. This is because the results of this study were conducted in Japan, where healthcare systems are well organized with minimal variations in sociodemographic conditions across regions.

The strength of this study is that we used large data sets and identified the association between social support and ambulance use in older people. We believe that future studies should analyze longitudinal data to identify causal relationships and find the association between disease type and treatment intensity in emergency departments after ambulance use and social support by using medical claims databases.

## Conclusion

This study revealed that the association between social support and ambulance use among older adults. Our results suggest that social support can be an important factor related to ambulance use. We suggest that policymakers should implement the interventions to enhance the provision of social support to reduce ambulance use among older people. We believe that this research can be used to make policies that improve the burden on emergency medical care. Based on the findings in this study, these policies would be aimed at decreasing ambulance use by increasing family support.

### Supplementary Information

**Additional file 1: Supplemental figure 1.** Participating Municipalities in Japan Gerontological Evaluation Study (JAGES) in 2019 (Japan Gerontological Evaluation Study (JAGES) repository (URL:https://www.jages.net/)). Municipalities that have participated in JAGES2019 are shown in red and those that have participated in the past are shown in blue.

**Additional file 2: Supplemental Table 1.** Characteristics of frequent ambulance users according to the frequency of ambulance call per year.

**Additional file 3:. Supplemental Table 2.** Variance inflation factor (VIF) of Model 2 and 3 in Table 2s and 3.

**Additional file 4: Supplemental Table 3.** Hosmer-Lemeshow test and the c statistic about Table 2. Supplemental Table 4 Hosmer-Lemeshow test and the c statistic about Table 3.

## Data Availability

The datasets generated and/or analyzed during the current study are available in the Japan Gerontological Evaluation Study (JAGES) repository (https://www.jages.net/). The datasets used and/or analyzed during the current study are available from the corresponding author on reasonable request. All data generated or analyzed during this study are included in this published article. The data that support the findings of this study are available from JAGES but restrictions apply to the availability of these data, which were used under license for the current study, and so are not publicly available. Data are however available from the authors upon reasonable request and with permission of JAGES.

## Abbreviations

JAGES: Japan Gerontological Evaluation Study
CI: confidence interval
OR: odds ratios
