# Supplementary material for: Association between social support and ambulance use among older people in Japan: an empirical cross-sectional study
Source: BMC Emerg Med. 2024 Mar 5;24:37. doi: 10.1186/s12873-024-00953-8 (PMC10913287; doi:10.1186/s12873-024-00953-8)
Supplement: Supplementary file 1 — Additional file 1: Supplemental figure 1. Participating Municipalities in Japan Gerontological Evaluation Study (JAGES) in 2019 (Japan Gerontological Evaluation Study (JAGES) repository (URL:https://www.jages.net/)). Municipalities that have participated in JAGES2019 are shown in red and those that have participated in the past are shown in blue. [file 12873_2024_953_MOESM1_ESM.pdf]

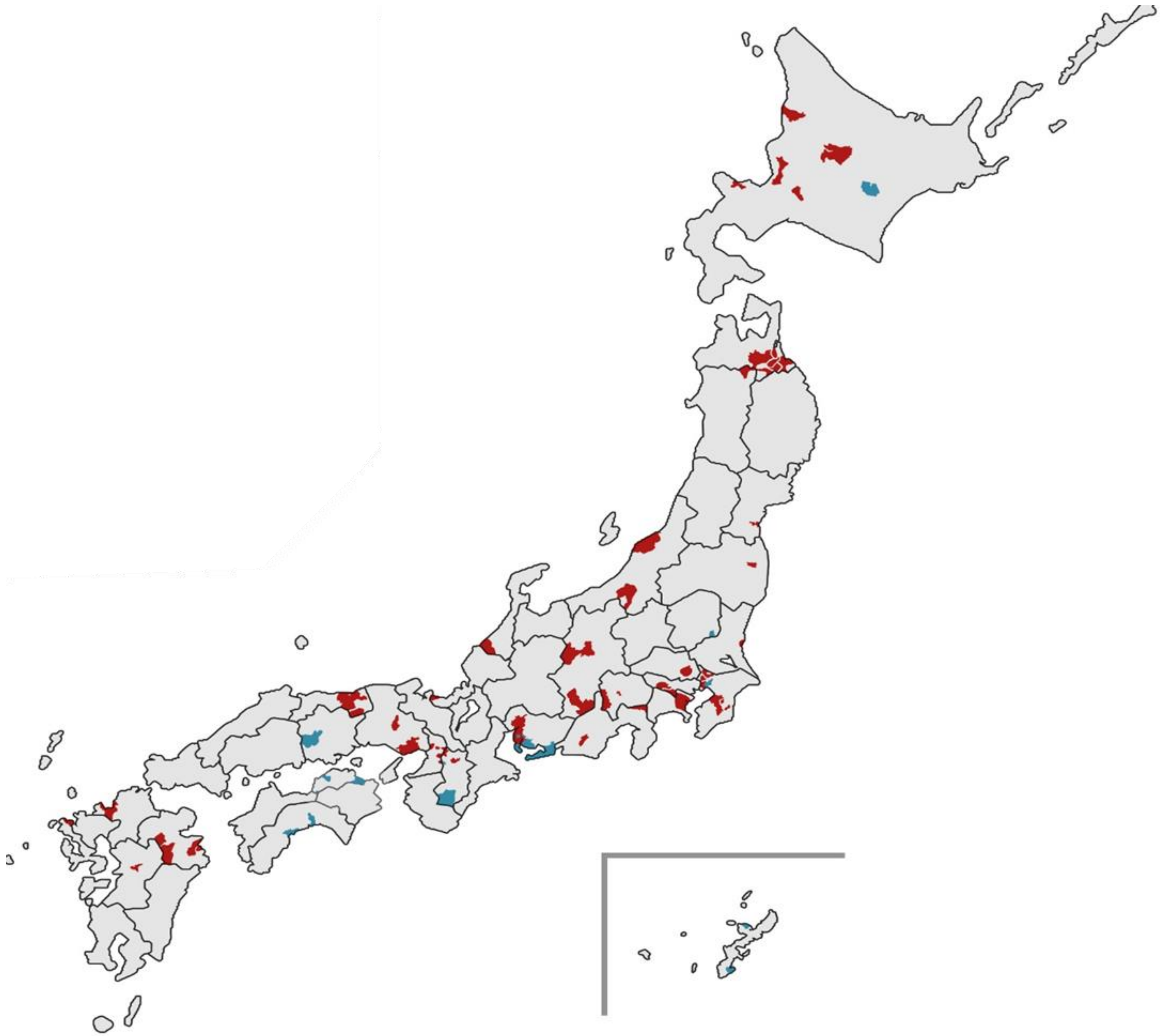

Supplemental figure1：Participating Municipalities in JAGES2019
(Japan Gerontological Evaluation Study (JAGES) repository (URL:https://www.jages.net/))
